# Complete Genome Sequence of the *Alfalfa latent virus*

**DOI:** 10.1128/genomeA.00250-15

**Published:** 2015-04-16

**Authors:** Lev G. Nemchinov, Jonathan Shao, Olga A. Postnikova

**Affiliations:** USDA-ARS, Beltsville Agricultural Research Center, Molecular Plant Pathology Laboratory, Beltsville, Maryland, USA

## Abstract

The first complete genome sequence of the *Alfalfa latent carlavirus* (ALV) was obtained by primer walking and Illumina RNA sequencing. The virus differs substantially from the Czech ALV isolate and the *Pea streak virus* isolate from Wisconsin. The absence of a clear nucleic acid-binding protein indicates ALV divergence from other carlaviruses.

## GENOME ANNOUNCEMENT

*Alfalfa latent virus* (ALV) is a member of the carlavirus group and occurs symptomlessly in alfalfa (*Medicago sativa*) (1). In the United States it is prevalent in Nebraska and Wisconsin (http://www.dpvweb.net/dpv/showdpv.php?dpvno=211). The virus is recognized as a strain of *Pea streak virus* (PeSV) (2–4). No complete genomic sequence of PeSV or ALV was available prior to this report.

ALV-infected tissues were obtained from ATCC (PV-264 isolate from Lancaster County, NE, USA). Total RNA was extracted with the TRIzol protocol and used in primer walking, employing SuperScript RT-PCR system (Life Technologies) for long templates. The 5′ end of the genome was amplified with the RACE system (Life Technologies). PCR fragments were sequenced and assembled into contigs, and the complete genome consensus sequence was generated.

Independently, total RNA purified with the RNeasy mini kit (Qiagen) was used as a template for Illumina RNA sequencing. Paired-end libraries (470 million reads) were cleaned by removing host plant DNA. Using Bowtie2 (5), the reads were mapped to the *Medicago truncatula* genome (Mt4.0v1), mitochondrial DNA, and plastid DNA. The 200 million reads that did not map were assembled with Trinity (6) into 73,574 contigs and queried against known plant viruses using BLASTn (7). A single contig was selected that had a negative *E* value with other plant viruses. It was 99.8% identical to the PCR-derived ALV sequence. The consensus sequence of 8,041 nucleotides (nt) was produced and used for further analysis.

The ALV genome contains five tentative open reading frames encoding a viral replicase (RdRp) (65 to 5,791 nt, 1,909 aa), triple gene block protein 1 (TGB1) (5,823 to 6,557 nt, 245 aa), TGB2 (6,535 to 6,849 nt, 105 aa), TGB3 (6,831 to 7,031 nt, 67 aa), and capsid protein (CP) (7,068 to 7,952 nt, 295 aa). The ALV does not appear to encode a clear 3′ proximal nucleotide acid-binding protein (NABP) that is typical for carlaviruses.

On the nucleotide level, ALV has 99%, 76%, and 79% identity with the partial sequences of the same ALV isolate PV-264 (presumably a variant, GenBank: AY037925), the Czech ALV isolate (HM107774.1), and the Wisconsin PeSV isolate (AF354652), respectively. The next closest species from the genus *Carlavirus*, *Shallot latent virus*, is 48.5% identical to the ALV (PASC tool [8]).

On the amino acid level (BLASTp), ALV RdRp was 91% identical to the partial RdRP sequence of HM107774.1 and ~43% to 50% identical to the RdRP of other carlaviruses. Identity scores for the TGB proteins were as follows: TGB1, 96% to AY037925, 80% to AF354652, 72% to HM107774.1, and ~34% to 45% to other carlaviruses; TGB2, 100% to AY037925, 84% to AF354652, 75% to HM107774.1, and ~42% to 52% to other carlaviruses; TGB3, 95% to AY037925, 65% to AF354652, 62% to HM107774.1, and ~44% to 50% to other carlaviruses. The CP was 99% identical to AY037925, 96% to HM107774.1, 95% to AF354652, and 45% to 57% to other carlaviruses. We propose that the sequenced ALV isolate from Nebraska differs substantially from the Czech ALV and Wisconsin PeSV isolates. The absence of clear NABP indicates that ALV is divergent from other members of the genus *Carlavirus*.

### Nucleotide sequence accession number. 

The sequence has been deposited in GenBank under the accession number KP784454.

## References

[1] Veerisetty V, Brakke MK 1977 Alfalfa latent virus, a naturally occurring carlavirus in alfalfa. Phytopathology 67:1202–1206. doi:10.1094/Phyto-67-1202.

[2] King AMQ, Adams MJ, Carstens EB, Lefkowitz EJ (ed) 2012 Virus taxonomy: classification and nomenclature of viruses: ninth report of the International Committee on Taxonomy of Viruses. Elsevier Academic, San Diego, CA.

[3] Hampton RO 1981 Evidence suggesting identity between alfalfa latent and pea streak viruses. Phytopathology 71:223.

[4] Edwardson GR, Christie RG 1991 Pea streak virus, p 108–111. In CRC handbook of viruses infecting legumes. CRC Press, Boca Raton, FL.

[5] Langmead B, Salzberg SL 2012 Fast gapped-read alignment with Bowtie 2. Nat Methods 9:357–359. doi:10.1038/nmeth.1923. 22388286 PMC3322381

[6] Grabherr MG, Haas BJ, Yassour M, Levin JZ, Thompson DA, Amit I, Adiconis X, Fan L, Raychowdhury R, Zeng Q, Chen Z, Mauceli E, Hacohen N, Gnirke A, Rhind N, di Palma F, Birren BW, Nusbaum C, Lindblad-Toh K, Friedman N, Regev A 2011 Full-length transcriptome assembly from RNA-seq data without a reference genome. Nat Biotechnol 29:644–652. doi:10.1038/nbt.1883. 21572440 PMC3571712

[7] Altschul SF, Gish W, Miller W, Myers EW, Lipman DJ 1990 Basic local alignment search tool. J Mol Biol 215:403–410. doi:10.1016/S0022-2836(05)80360-2. 2231712

[8] Bao Y, Chetvernin V, Tatusova T 2014 Improvements to pairwise sequence comparison (PASC): a genome-based web tool for virus classification. Arch Virol 159:3293–3304. doi:10.1007/s00705-014-2197-x. 25119676 PMC4221606

